# Supplementary material for: A novel SLC2A10 gain-of-function variant links glycolytic macrophage polarization to chronic nonbacterial osteomyelitis
Source: Life Sci Alliance. 2026 Jun 3;9(8):e202603772. doi: 10.26508/lsa.202603772 (PMC13234206; doi:10.26508/lsa.202603772)
Supplement: Supplementary file 2 [file LSA-2026-03772_TableS1.docx]

Table S1. 15 candidate variants related to CNO identified in a familial case after applying filtering criteria.

| Gene | Zygosity | Genomic Position | CDNA change | Protein change | ACMG category |
| --- | --- | --- | --- | --- | --- |
| DPYD | Het | chr1:97700547 | c.C2303A | p.T768K | Likely Pathogenic |
| ALK | Het | chr2:29917736 | c.G932A | p.R311H | Likely Pathogenic |
| PPID | Het | chr4:159632000 | c.G895C | p.A299P | Likely Pathogenic |
| PCDHB1 | Het | chr5:140431179 | c.G124C | p.G42R | Likely Pathogenic |
| SDK1 | Het | chr7:4259801 | c.G1061A | p.R354Q | Likely Pathogenic |
| FRMPD1 | Het | chr9:37733513 | c.C1039T | p.R347X | Likely Pathogenic |
| IQSEC3 | Het | chr12:250396 | c.A1189T | p.M397L | Likely Pathogenic |
| PFKM | Het | chr12:48525134 | c.C195G | p.I65M | Likely Pathogenic |
| SLC2A10 | Het | chr20:45354551 | c.C876A | p.D292E | Likely Pathogenic |
| SYCP1 | Het | chr1:115486960 | c.G1927T | p.V643F | Pathogenic |
| COL5A2 | Het | chr2:189922044 | . | . | Pathogenic |
| ADAMTS6 | Het | chr5:64747447 | c.C928G | p.P310A | Pathogenic |
| NT5E | Het | chr6:86197051 | . | . | Pathogenic |
| MED23 | Het | chr6:131941871 | . | . | Pathogenic |
| GALC | Het | chr14:88414224 | . | . | Pathogenic |
