# Supplementary material for: A novel SLC2A10 gain-of-function variant links glycolytic macrophage polarization to chronic nonbacterial osteomyelitis
Source: Life Sci Alliance. 2026 Jun 3;9(8):e202603772. doi: 10.26508/lsa.202603772 (PMC13234206; doi:10.26508/lsa.202603772)
Supplement: Supplementary file 3 [file LSA-2026-03772_TableS2.docx]

Table S2. Primers used for cloning and site-directed mutagenesis of mouse Slc2a10

|  | In-Fusion cloning | Site-directed mutagenesis |
| --- | --- | --- |
| F | AGGTCGACTCTAGAGGATCCCGCCACCATGGGCCTTCGCCCAGCTGTCCTCCTGCTCTGTGCC | GCTGGTGGAACGTGCAGGCCGCAG |
| R | TCCTTGTAGTCCATACCGGTGGAGGCTGAGGAGACATCCAGGC | GGCCTGCACGTTCCACCAGCCCGT |
