# Supplementary material for: A novel SLC2A10 gain-of-function variant links glycolytic macrophage polarization to chronic nonbacterial osteomyelitis
Source: Life Sci Alliance. 2026 Jun 3;9(8):e202603772. doi: 10.26508/lsa.202603772 (PMC13234206; doi:10.26508/lsa.202603772)
Supplement: Supplementary file 4 [file LSA-2026-03772_TableS3.docx]

Table S3. Primers used for the sanger sequencing in this study.

| SLC2A10-F | 5-GGATGGATAAATGAAGGTGT or  5-CAACTATGCACTGGCTGGTA |
| --- | --- |
| SLC2A10-R | 5-AACATAAAATTAGGCACTGG |
