# Supplementary material for: A novel SLC2A10 gain-of-function variant links glycolytic macrophage polarization to chronic nonbacterial osteomyelitis
Source: Life Sci Alliance. 2026 Jun 3;9(8):e202603772. doi: 10.26508/lsa.202603772 (PMC13234206; doi:10.26508/lsa.202603772)
Supplement: Supplementary file 5 [file LSA-2026-03772_TableS4.docx]

Table S4. Primers used for the RT-qPCR assay in this study

| Species | Genes | Forward primers (5’-3’) | Reverse primers (5’-3’) |
| --- | --- | --- | --- |
| Fish | *mmp9* | CAGAGAGGAAAAGGCAAGGTG | AGGAATACATCATGTGAATCAATG |
| Fish | *alp* | AGAGAAGCGGCCTGATTACT | ACACCCATCCCATCTCCAAG |
| Fish | *ctsk* | CTATAAAGAGATTCCTCAGGG | ACACGGGTCCCACATTGG |
| Fish | *rankl* | CTCACCTTCCAATCAAGACGCCC | CTTTCATGCCATCCCAGGCTATCT |
| Fish | *trap* | CCCCATAGAGACCGCTACAG | ACATCAGTGACGCCCTTGTA |
| Fish | *il1b* | ATCAAACCCCAATCCACAGAGT | GGCACTGAAGACACCACGTT |
| Fish | *tnfα* | TCACGCTCCATAAGACCCAG | GATGTGCAAAGACACCTGGC |
| Fish | *β-actin* | ACAGGGAAAAGATGACACAGATCA | CAGCCTGGATGGCAACGTA |
| Fish | *il8* | TGTTTTCCTGGCATTTCTGACC | TTTACAGTGTGGGCTTGGAGGG |
| Fish | *il10* | TCACGTCATGAACGAGATCC | CCTCTTGCATTTCACCATATCC |
| Mouse | *Ctsk* | CTCGGCGTTTAATTTGGGAGA | TCGAGAGGGAGGTATTCTGAGT |
| Mouse | *Rank* | CCAGGAGAGGCATTATGAGCA | ACTGTCGGAGGTAGGAGTGC |
| Mouse | *Calcr* | GAGGTTCCTTTCGTGAACAG | AGTCAGTGAGATTGGTAGGAGC |
| Mouse | *Trap* | CACTCCCACCCTGAGATTTGT | CCCCAGAGACATGATGAAGTCA |
| Mouse | *Dc-stramp* | GGGGACTTATGTGTTTCCACG | ACAAAGCAACAGACTCCCAAAT |
| Mouse | *Mmp9* | GCAGAGGCATACTTGTACCG | TGATGTTATGATGGTCCCACTTG |
| Mouse | *Nfact1* | GGAGAGTCCGAGAATCGAGAT | TTGCAGCTAGGAAGTACGTCT |
| Mouse | *Il4* | GGTCTCAACCCCCAGCTAGT | GCCGATGATCTCTCTCAAGTGAT |
| Mouse | *Il10* | CTTACTGACTGGCATGAGGATCA | GCAGCTCTAGGAGCATGTGG |
| Mouse | *Tgfβ* | CCACCTGCAAGACCATCGAC | CTGGCGAGCCTTAGTTTGGAC |
| Mouse | *Alp* | CCAACTCTTTTGTGCCAGAGA | GGCTACATTGGTGTTGAGCTTTT |
| Mouse | *Col1* | GCTCCTCTTAGGGGCCACT | ATTGGGGACCCTTAGGCCAT |
| Mouse | *Il6* | CTGCAAGAGACTTCCATCCAG | AGTGGTATAGACAGGTCTGTTGG |
| Mouse | *Il1β* | GAAATGCCACCTTTTGACAGTG | TGGATGCTCTCATCAGGACAG |
| Mouse | *Cxcl15* | TCGAGACCATTTACTGCAACAG | CATTGCCGGTGGAAATTCCTT |
| Mouse | *iNos* | GTTCTCAGCCCAACAATACAAGA | GTGGACGGGTCGATGTCAC |
| Mouse | *Slc2a10* | GGGCCTGACCTTCGGATATG | GCTCCTGTTCGAGGCAACT |
| Mouse | *Runx2* | GACTGTGGTTACCGTCATGGC | ACTTGGTTTTTCATAACAGCGGA |
| Mouse | *Bgp* | CTGACCTCACAGATCCCAAGC | TGGTCTGATAGCTCGTCACAAG |
| Mouse | *Opg* | CCTTGCCCTGACCACTCTTAT | CACACACTCGGTTGTGGGT |
| Mouse | *Sp7* | GGAAAGGAGGCACAAAGAAGC | CCCCTTAGGCACTAGGAGC |
| Mouse | *Tnfα* | CAGGCGGTGCCTATGTCTC | CGATCACCCCGAAGTTCAGTAG |
