# Supplementary material for: A novel SLC2A10 gain-of-function variant links glycolytic macrophage polarization to chronic nonbacterial osteomyelitis
Source: Life Sci Alliance. 2026 Jun 3;9(8):e202603772. doi: 10.26508/lsa.202603772 (PMC13234206; doi:10.26508/lsa.202603772)
Supplement: Supplementary file 6 [file LSA-2026-03772_TableS5.docx]

Table S5. Antibodies for Western Blot Experiments

| Antibody Target | Vendor | Catalog Number | Dilution |
| --- | --- | --- | --- |
| β-actin | ZSGB-BIO | TA-09 | 1：2000 |
| SLC2A10 | Proteintech | 20405-1-AP | 1：1000 |
| RANKL | Abcam | ab124797 | 1：1000 |
| CTSK | Abcam | ab19027 | 1：1000 |
| NFATC1 | Proteintech | 66963-1-Ig | 1：1000 |
| MMP9 | CST | 24317 | 1：1000 |
| iNOS | CST | 13120T | 1：1000 |
| NF-κB p65 | CST | 8242S | 1：1000 |
| Phospho-NF-κB p65 | CST | 3033S | 1：1000 |
| PFKFB3 | Proteintech | 13763-1-AP | 1：2000 |
| LDHA | Proteintech | 19987-1-AP | 1：2000 |
| HK2 | CST | 2867S | 1：1000 |
| PFKP | Abcolnal | A7916 | 1：1000 |
