# Supplementary material for: A novel SLC2A10 gain-of-function variant links glycolytic macrophage polarization to chronic nonbacterial osteomyelitis
Source: Life Sci Alliance. 2026 Jun 3;9(8):e202603772. doi: 10.26508/lsa.202603772 (PMC13234206; doi:10.26508/lsa.202603772)
Supplement: Supplementary file 7 [file LSA-2026-03772_TableS6.docx]

Table S6. Zebrafish embryo mRNA injection concentration test

| mRNA concentration | Deaths/Number of embryos injected | Abnormal/Number of embryos injected |
| --- | --- | --- |
| 100ng/µL | 9/130 | 24/130 |
| 200ng/µL | 18/120 | 28/120 |
| 400ng/µL | 20/120 | 33/120 |
